# Appendiceal Inversion Presenting as a Cecal Polypoid Mass on Screening Colonoscopy: A Case Report and Review of Available Diagnostic Adjuncts to Differentiate Benign From Malignant Colorectal Pathology

**DOI:** 10.7759/cureus.35645

**Published:** 2023-03-01

**Authors:** Anna Axentiev, Bushra Shehzad, Irina Bernescu

**Affiliations:** 1 General Surgery, Ascension Saint Agnes Hospital, Baltimore, USA; 2 General Surgery, Ross University School of Medicine, Baltimore, USA; 3 Colorectal Surgery, Ascension Saint Agnes Hospital, Baltimore, USA

**Keywords:** appendix stump, screening colonoscopy, colonoscopy and polypectomy, colorectal polyps, colorectal cancer

## Abstract

Appendiceal inversion is uncommon. It may be a benign finding or seen in association with malignant pathology. When detected, it masquerades as a cecal polyp which poses a diagnostic dilemma with malignancy in the differential. In this report, we highlight a case of a 51-year-old patient with an extensive surgical history as a newborn in the setting of omphalocele and intestinal malrotation, who was found to have a 4 cm cecal polypoid growth on screening colonoscopy. He underwent a cecectomy for tissue diagnosis. Ultimately, the polyp was found to be an inverted appendix without evidence of malignancy. Currently, suspicious colorectal lesions which cannot be removed by polypectomy are primarily addressed with surgical excision. We reviewed the literature for available diagnostic adjuncts to better differentiate benign from malignant colorectal pathology. The application of advanced imaging and molecular technology will allow for improved diagnostic accuracy and subsequent operative planning.

## Introduction

Appendiceal inversion is uncommon, seen in up to 1.5% of colonoscopies, and presents a diagnostic challenge [1]. It can arise via intussusception or as a result of intentional stump inversion during an open appendectomy [2]. Appendiceal inversion can mimic or represent a pathological process, thus warranting further investigation. An appendectomy is one of the most frequently performed abdominal surgical procedures. A common operative approach in open appendectomy, especially in pediatric patients, involves ligation and inversion of the appendiceal stump into the cecum. The proposed advantages of stump inversion include double closure of the cecal wall, decreased contamination from an intra-peritoneal stump, and minimized risk of adhesions [3]. However, there are reports of an appendiceal stump harboring a neoplasm [4-5]. As a result, appendiceal inversion, or what appears as a cecal polyp at the appendiceal orifice on imaging or colonoscopy, must be further investigated and differentiated from a pathologic process.

Several factors could help differentiate benign from malignant cecal polyps. Ileocecal thickening greater than 3 mm is considered abnormal and characteristic of the inflammatory or neoplastic process [6]. Calcifications can also indicate neoplastic pathology. Colorectal and ovarian neoplasms commonly manifest with such calcifications although this pathophysiology is heterogeneous and poorly understood [7]. Colorectal pathology can be identified using various screening modalities, such as colonoscopy, computed tomographic colonography (CTC), and fecal, immunochemical, and multitarget stool DNA tests. This report highlights the case of a 51-year-old male patient with a history of omphalocele and intestinal malrotation who was found to have a cecal polypoid structure on screening colonoscopy. He underwent cecal resection and the final pathology of the surgical specimen revealed a benign inverted appendix. Currently, suspicious colorectal lesions which cannot be removed by polypectomy are predominantly addressed by surgical excision. We reviewed and highlighted available diagnostic adjuncts that may help differentiate benign from malignant colorectal pathology at present, and in the future to further individualize and select those patients who will benefit from surgical excision of suspicious colorectal lesions.

## Case presentation

**Figure 1:**
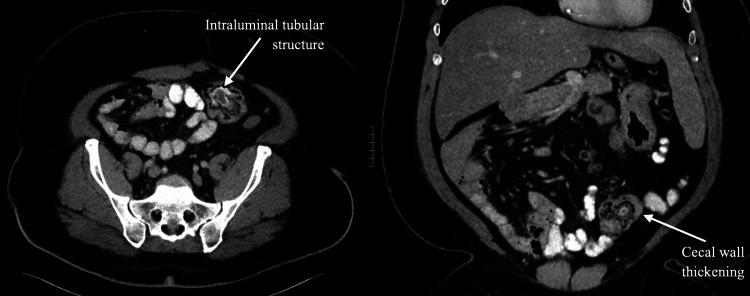
The CT of the abdomen and pelvis demonstrating peripherally calcified 4.5 cm tubular structure protruding into the cecal lumen with cecal wall thickening. Notably, the cecum is found in the left lower quadrant.

This report describes the case of a 51-year-old male patient with a history of omphalocele and intestinal malrotation, requiring multiple abdominal surgeries including appendectomy, as a newborn. He was found to have a cecal mass on screening colonoscopy. This finding was followed by CT demonstrating a 4.5 cm peripherally calcified tubular structure within the lumen of the cecum with associated cecal wall thickening (Figure 1).

**Figure 2:**
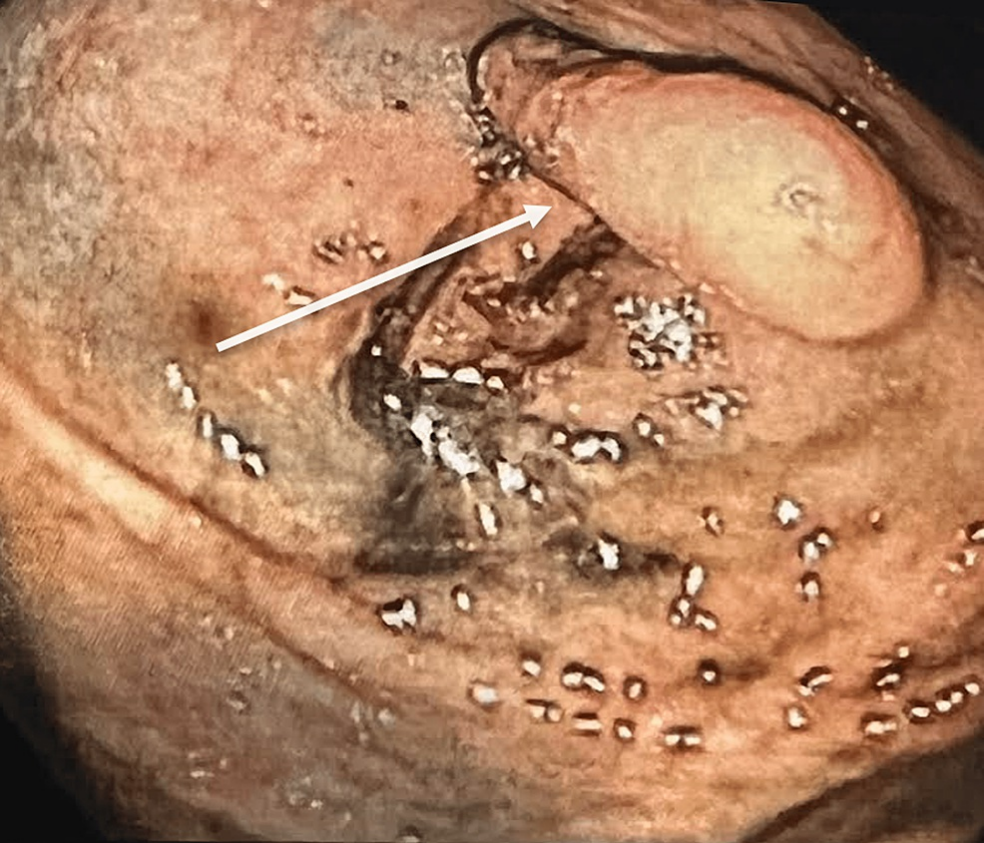
Polypoid mass at the appendiceal orifice on colonoscopy

Although an intraluminal appendix was suspected, neoplasm could not be ruled out. The patient was referred to a colorectal surgeon for further management. The patient was noted to experience non-specific abdominal pain with no evidence of gastrointestinal bleeding. There was no family history of colon cancer. Surgical resection was discussed to obtain tissue diagnosis and rule out malignancy. A preoperative colonoscopy was performed and re-demonstrated a solid, non-compressible, elongated, 2 cm lesion, protruding into the cecal lumen from the level of the appendiceal orifice (Figure 2). Colonoscopic biopsies were forgone to minimize the risk of perforation and the base of the lesion was tattooed to facilitate the surgical resection.

**Figure 3:**
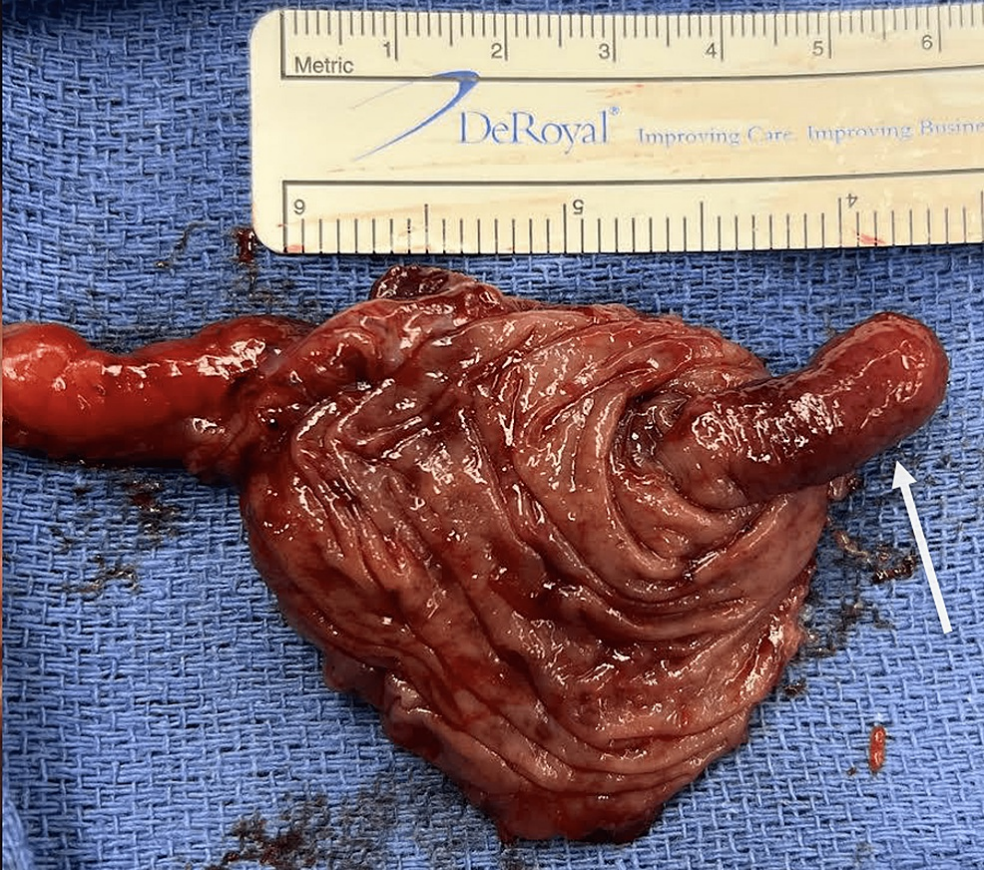
Cecectomy specimen with intraluminal mass

Due to his anatomical variation from intestinal malrotation and extensive post-surgical adhesions, the patient underwent a robotic converted-to-open cecectomy via transverse left lower quadrant incision (specimen seen in Figure 3). The pathology report described an intraluminal polyp consistent with an inverted appendix, with no dysplasia or neoplasia. The patient’s postoperative course was uncomplicated.

## Discussion

All-cause appendiceal inversion is a rare finding that is seldom reported in the literature [1]. Appendiceal inversion may arise via intussusception or by ligation and stump inversion technique, frequently performed during open appendectomy, especially in pediatric patients. This report highlights the case of a 51-year-old male patient with a history of omphalocele and intestinal malrotation, requiring multiple abdominal surgeries including appendectomy as a newborn, who was found to have a cecal polypoid structure on screening colonoscopy. With malignancy on the differential, the patient underwent cecal resection for tissue diagnosis and the final pathology of the surgical specimen revealed a benign inverted appendix. Although appendiceal inversion may be benign, as in our patient’s case, it may harbor appendiceal mucinous neoplasms, neuroendocrine tumors, and other malignant pathology with an incidence of less than 1% [4,5,8,9]. Imaging findings suggestive of malignancy include ileocecal thickening greater than 3 mm and calcifications [5,6]. As such, further diagnostic steps should be undertaken to rule out malignancy and guide surgical management in cases of suspected appendiceal inversion. Here, we discuss current and future diagnostic adjuncts to differentiate benign from malignant colorectal pathology.

Available modalities for colorectal cancer (CRC) screening include colonoscopy, computed tomographic colonography (CTC), as well as stool-based tests including fecal immunochemical and multitarget DNA tests. While colonoscopy remains the gold standard for CRC screening, newer detection modalities with advanced imaging, endoscopy, molecular technology and artificial intelligence (AI) / machine learning (ML) may improve diagnostic accuracy.

The technique most comparable to colonoscopy in detecting advanced colorectal neoplasia, with a sensitivity of 96.1%, is CTC [10]. Computed tomographic colonography is less invasive than a colonoscopy and polyps greater than 6 mm detected utilizing this technique are usually recommended to undergo polypectomy to rule out malignancy. Other important features of advanced neoplasia such as high-grade or villous histology are not assessed by conventional CTC. Volumetric textural analysis (VTA) utilizes the ability of CT to resolve different density arrangements to quantitatively evaluate the texture of colorectal polyps. Certain volumetric textural features are predictive of polyp histology. The VTA demonstrated similar accuracy to human readers in differentiating between benign and malignant lesions ≥3 cm [11]. Further investigation and application of this technology in smaller lesions could help select those patients that should undergo endoscopic or operative intervention.

Advanced endoscopy applications also show significant promise in differentiating benign from malignant polyps. Techniques such as non-selective fluorophore indocyanine green (ICG) with near-infrared (NIR) technology allow for enhanced real-time assessment of tissue microperfusion. Malignant lesions demonstrate dynamic curves which peak significantly later compared to benign lesions, which demonstrate a faster intensity drop [12]. Additionally, combined molecular and fluorescent endoscopy may be utilized to detect CRC. An example is the c-Met-targeted fluorescent peptide [13]. This was administered intravenously and revealed significantly higher fluorescence in the colorectal lesions than in surrounding tissue, correlating to c-Met overexpression [13]. Similar findings were achieved using antibodies as targeting agents [14]. Other molecular targets currently under investigation include carcinoembryonic antigen (CEA), cyclooxygenase 2 (PTGS2), and Thomsen-Friedenreich antigen.

An emerging area of research involves AI-assisted colonoscopy for colorectal polyp detection and characterization. This utilizes computer-aided detection (CAD), an artificial neural network and deep learning technique that is highly effective at performing medical image analysis. Multiple CADs have been developed and are being studied for colorectal polyp detection and characterization. For instance, improved adenoma detection rate within small polyps using CAD during real-time colonoscopy has been previously demonstrated [15]. Artificial intelligence can be applied to assist in characterizing colorectal lesions such as those seen with VTA in CTC as well as other applications including advanced endoscopic imaging utilizing chromoendoscopy using dye spray (indigo carmine or crystal violet) for polyp surface pit pattern, histological differentiation via endocytoscopy, laser-induced fluorescence spectroscopy (LIFS), and autofluorescence imaging (AFI) [16]. Such advances allow additional scrutiny of tissues in the diagnosis of CRC.

## Conclusions

To this day, colonoscopy remains the gold standard modality for CRC screening with biopsy and polypectomy performed for diagnosis. Albeit rare, an inverted appendix can masquerade as a cecal polyp at the appendiceal orifice and may harbor appendiceal mucinous neoplasms, neuroendocrine tumors, and other malignancies. Currently, suspicious colorectal lesions which cannot be removed by polypectomy due to large size or risk of bowel perforation, such as the one seen in our patient, are primarily addressed with surgical excision. This approach is invasive and carries the risk of bleeding, infection, damage to nearby structures, and more. The reviewed diagnostic adjuncts which include advanced imaging, endoscopy, molecular technology, and AI/ML may improve diagnostic accuracy to better differentiate benign from malignant colorectal pathology. Application of such adjuncts in the future will help further categorize suspicious colorectal lesions that would otherwise require polypectomy or surgical excision. This paves the way for less invasive methods of CRC screening and selecting those patients who would further benefit from surgery.
